# Quantitative evaluation of transdermal drug delivery patches on human skin with *in vivo* THz-TDS

**DOI:** 10.1364/BOE.473097

**Published:** 2023-02-15

**Authors:** Xuefei Ding, Gonçalo Costa, A. I. Hernandez-Serrano, Rayko I. Stantchev, Gabit Nurumbetov, David M. Haddleton, Emma Pickwell-MacPherson

**Affiliations:** 1 Department of Physics, University of Warwick, Gibbet Hill Road, Coventry, CV4 7AL, UK; 2 Medherant Ltd., The Venture Centre, University of Warwick Science Park, Coventry, CV4 7EZ, UK; 3 Department of Chemistry, University of Warwick, Gibbet Hill Road, Coventry, CV4 7AL, UK

## Abstract

Transdermal drug delivery (TDD) has been widely used in medical treatments due to various advantages, including delivering drugs at a consistent rate. However, variations in skin hydration can have a significant effect on the permeability of chemicals. Therefore, it is essential to study the changes in skin hydration induced by TDD patches for better control of the delivery rate. In this work, *in vivo* terahertz (THz) spectroscopy is conducted to quantitatively monitor human skin after the application of patches with different backing materials and propylene glycol concentrations. Changes in skin hydration and skin response to occlusion induced by other patches are investigated and compared. Our work demonstrates the potential application of *in vivo* THz measurements in label-free, non-invasive evaluation of transdermal patches on human skin and further reveals the mechanism behind the effect.

## 1. Introduction

Transdermal drug delivery (TDD) has become a preferable alternative to conventional methods such as oral delivery and hypodermic injection due to being non-invasive, self-administered, inexpensive and reducing over-dose situations [1,2]. So far, TDD has been utilized in medical treatments for delivering drugs including nicotine, fentanyl and scopolamine [3].

When designing the patches for TDD, the choice of excipient composition and backing material are influential factors to consider. Permeation enhancers are often added to TDD patches to enhance the penetration of the drug through the skin and temporarily decrease the resistance of the skin barrier [4]. Propylene glycol (PG) is a commonly chosen permeation enhancer which also serves as humectant and co-solvent for poorly soluble chemicals, and it has been reported to have stronger ability in improving the transdermal flux for topical drug delivery than other widely used enhancers [5,6]. Backing materials can alter the drug delivery efficacy and the response of skin to the patch through different levels of occlusive features. When the drug contained in the patch is highly potent and toxic, it is essential to prevent molecular exchange with the environment by using a fully occlusive backing [7]. However, long-term occlusion of the skin can cause over-accumulation of water at the skin surface without evaporation and lead to skin irritation, therefore a partially occlusive backing is preferred under certain circumstances [8].

One of the most preferable advantages of TDD patches is that they allow a controlled amount of drugs to be delivered into the human skin at a relatively consistent rate. However, studies have noted that the hydration of the skin area to be treated is an important factor affecting the drug delivery rate, with the permeability of skin increasing significantly with the growth in hydration [9]. The stratum corneum (SC) is the outermost layer of skin, which has a thickness of approximately 10-15µm in the dry state and can swell up-to 40µm when hydrated; by increasing the hydration level of SC, the barrier function of skin can be reduced [3]. Therefore, it is essential to study the influence of TDD patches on skin hydration in order to have better control and understanding of the drug delivery rate. Additionally, analysing the recovery of skin after removing the patches can provide supporting information on the frequency of applying patches and switching the application area [10].

Terahertz (THz) radiation is a novel technique for biomedical examinations with the assets of non-ionizing photon energies and high sensitivity to water [11]. The potential use of *ex vivo* THz spectroscopy and imaging for studying the contrast between healthy and cancerous tissues [12–14] and to quantify glucose levels in diabetic blood plasma [15,16] has been investigated; subsequently, approaches to enhance THz characterization sensitivity of biological tissues and solutions have been proposed [17,18]. Recently, *in vivo* THz measurements have aroused increasing interest for its advantage in non-invasively monitoring the response of living skin to certain treatments. Studies have revealed the potential for using THz time-domain spectroscopy (THz-TDS) and THz imaging to assess the depth and severity of burn wounds *in vivo* [19,20]. Research has also been conducted on *in vivo* THz sensing of corneal tissue hydration [21,22] and THz imaging for *in vivo* evaluation of diabetic foot syndrome [23].

THz spectroscopic and imaging techniques have recently been utilized as non-destructive, label-free methods for evaluation of transdermal drug delivery. Kim et al. have demonstrated the ability of THz dynamic imaging in visualizing the spatial distribution and penetration of a topical drug on excised mouse skin [24]. Later on, Wang et al. applied *ex vivo* THz imaging to compare the efficacy between different TDD methods including the use of microneedles and nanoneedles [25]. Lee et al. reported a new way of quantifying drug delivery rate with THz sensing by measuring nicotine patches before and after application [26]. The above-mentioned studies are either conducted *ex vivo* on excised skin tissues or verified indirectly by measuring the changes on the patches. To acquire a more comparable result to the actual dynamic process in skin during TDD, *in vivo* THz measurements on human skin need to be studied. Lindley-Hatcher et al. have conducted *in vivo* THz point scan on volar forearm to investigate the changes induced in the skin by TDD patches with different backing materials and PG concentrations [10]. The study focused on how different types of patches can influence the drug delivery rate through the changes in skin hydration and skin’s response to occlusion. Their work provided a proof of concept with a small scale of participants and measured with THz point scan observing only a single location on the skin.

In this study, we developed the work further by increasing the number of subjects measured and within a larger scale we were able to categorize them into different skin groups according to their original skin condition. We have investigated the effect of different types of patches on the skin hydration level and skin occlusion process [27]; trends were studied in skin type groups as well as in general for all subjects, offering a deeper understanding of the skin’s response to the patches. In addition to the *in vivo* THz point scan, we also conducted *in vivo* THz imaging and 3D camera imaging on skin to visualize the results. THz imaging provided spatial information of the skin hydration, while the 3D camera imaging showed changes in the roughness of skin before and after patch application. Both techniques provided supplementary information to THz-TDS revealing the mechanism of the changes induced in the skin by TDD patches.

## 2. Methodology

### 2.1 THz system and other experimental setup

**Fig. 1.**
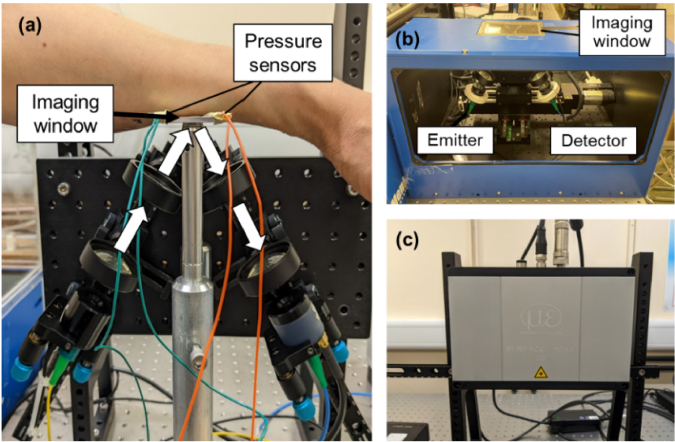
(a) Menlo TERA K15 THz spectrometer set up in reflection geometry at an angle of 30°; (b) K15 imaging system; (c) surfaceCONTROL 3D 3500 sensor from Micro Epsilon.

In this study, *in vivo* THz skin measurements were acquired with a Menlo TERA K15 THz Time-Domain system. The system was set up in reflection geometry with a THz emitter and detector vertically assembled on optical rails at an incident angle of 30° to the quartz imaging window (Fig. 1(a)). To measure the THz response of living skin, each participant was asked to rest their volar forearm on top of the quartz imaging window as shown in Fig. 1(a) for one minute. During that time the THz system recorded approximately 280 reflected THz pulses from a single point on the skin. For the *in vivo* THz imaging, the K15 spectrometer was connected to an identical reflection setup on a motorized x-y imaging stage (Fig. 1(b)) for raster scanning the skin. Each image has a size of 17mm × 17 mm covering the full area where the patches were applied, taking approximately 3 min to image the area with a resolution of 1 mm in both x-y directions. A reference and baseline measurement are also taken at each point such that the processed waveform (Eq. (1)) is calculated individually for each point in the image to account for any inhomogeneity in the imaging window. A high-precision surfaceCONTROL 3D 3500 by Micro-Epsilon camera was used to take the 3D images of the skin (Fig. 1(c)), providing information about the skin surface roughness. The 3D camera can achieve a 2.2 million cloud points per second with a vertical resolution down to 1.0 µm and an x-y resolution of 40 µm.

### 2.2 Materials and protocol for skin measurements

Ethical approval was obtained for this study from the Biomedical Scientific Research Ethics Committee, BSREC, (REGO-2018-2273 AM03). The 19 subjects participating in this study were in the age group of 23 to 33 and possessed healthy skin in the regions of interest. Prior to the start of the study, all subjects were informed of the experiments and gave their signed consent. To provide further insight into the significance of the observations in the previous work [10] on the effects of the patches on the skin, woven and film based patches were applied on the volar forearms of the 19 subjects.

The purpose of this study was to explore the changes that transdermal drug delivery patches generate in the skin via *in vivo* THz spectroscopy. As previously stated, in this study patches with two types of backing materials were used: the fully occlusive poly(ethylene terephthalate (PET) film-backed patches and the partially occlusive woven-backed patches; and for each type of patch, an excipient with propylene glycol added at a concentration of 0%, 3% and 6% was tested. These are commonly used for the enhancement of TDD rate in such patches, and 10% transcutol was added in all patches as co-solvent. No drugs were present in the patches to allow singling out the changes induced on the skin to be a consequence of the patch material and of the excipient concentration.

**Fig. 2.**
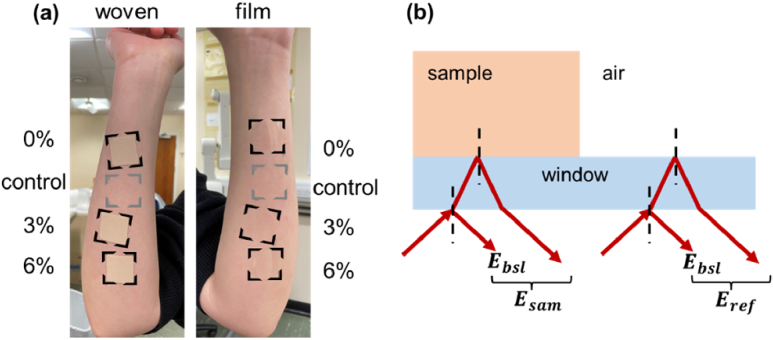
(a) Photo of the areas of interest indicating the locations of patches on the volar forearms. (b) A schematic diagram of the reflected signals of bulk sample in reflection geometry.

Figure 2(a) shows three woven-backed patches and three film-backed patches applied to the right and left volar forearm respectively; a control area was also marked on each arm to take into account the natural variation of skin occurring between each set of measurements. All of the 8 areas were marked with surgical skin marking pen in advance to keep track of the areas treated with patches. The measurements were taken in identical time frames: before the application of the patches, then 0 minutes, 30 minutes and 4 hours after removing the patches. Patches were applied and removed in a fixed order to allow each patch being applied on the skin for 24 hours. The ‘0 min’ measurements were immediately conducted after removing each patch, and then the skin areas were measured at 30 min and 4 hours after the time of removal in the same order as patches were removed. The control areas were measured each time along with the treated areas.

To further assure repeatability in the measurements, an established protocol for THz *in vivo* skin measurements was followed, and the pressure of skin contact and environment condition factors were taken into consideration [28]. Previous studies have found that the pressure between the skin and the imaging window affects the THz response of the sample [29]. To mitigate against this effect, pressure sensors were applied on each side of the quartz window to assure the pressure applied on the window was kept constantly in a range of 1.5-2.5 N/cm2. All participants were subjected to a 20-minute period of acclimatization in the lab before starting measurements; and other external factors that might affect the skin properties of each individual were accounted for through data processing.

### 2.3 Data processing

*In vivo* THz measurements of volar forearm are conducted in reflection geometry with an imaging window in contact with the skin. In this case the detected sample signal $E_{sam}$ contains two pulses (Fig. 2(b)): the first pulse is the reflection from the air-window interface which is defined as the baseline $E_{bsl}$ ; and the second pulse is the actual reflection from the window-sample interface. The reflection from the bare window is also measured as the reference signal $E_{ref}$ . $E_{sam}$ , $E_{bsl}$ and $E_{ref}$ are all functions of time, *t*, which is the optical delay in picoseconds (ps). Due to the ringing effect, the baseline needs to be subtracted from the sample and reference signals (we measured the baseline signal from a very thick window where we only see the first reflection). The reference and the baseline were measured on each day during the study to eliminate the subtle variation in system signals. The processed signal is then calculated according to Eq. (1), where a double Gaussian filter is applied to remove noise [30,31].

(1) $$processed\;signal=iFFT\left(FFT(filter)\times \frac{FFT(E_{sam}(t)-E_{bsl}(t))}{FFT(E_{ref}(t)-E_{bsl}(t))}\right)$$

**Fig. 3.**
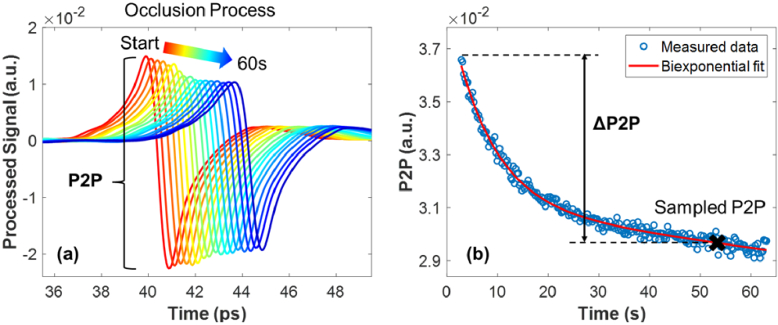
(a) An example of the processed THz signals measured from untreated skin for 60s of occlusion time. The pulses are plotted every half second for the first two seconds and every five second for the rest; they have been shifted horizontally for clarity. Peak-to-peak (P2P) index is defined. (b) The correlation between the P2P of processed signal and the occlusion time. A biexponential function is fitted to the measured data. A sampled point is taken at 52s into occlusion and the definition of ΔP2P is illustrated.

Figure 3(a) presents the processed THz signals throughout a measurement of untreated skin occluded for 60s in which the signals were shifted horizontally for clarity. The peak-to-peak (P2P) variable is defined as the change between the highest and lowest amplitude of the processed signal. A decrease in P2P is observed for the 60s process which was proposed in a previous study as the occlusion effect: when skin is in contact with the quartz imaging window, the surface of skin is occluded leading to accumulation of water, which will then reduce the reflection at quartz-skin interface [32]. Figure 3(b) is the occlusion curve showing how the P2P changes with occlusion time and the data points are fitted with a biexponential function. Here we take a sampled point on the biexponential fit at 52s into occlusion when the curve tends to be stable, and the ΔP2P variable is defined as the difference between the first point into occlusion and the sampled point.

In this study, we measure the skin at different time points throughout a rather long scale of time (28 hours), so it is necessary to consider the natural variation of skin while investigating the effects induced by the patch application. Therefore, the normalized relative change (NRC) is calculated to isolate the change of the THz response of skin that is caused by the patches [28]:

(2) $$NRC(\%)=\frac{\left(X_{Tt}-X_{T0})-(X_{Ct}-X_{C0}\right)}{X_{T0}+(X_{Ct}-X_{C0})}\times 100$$

where $X_{Tt}$ and $X_{Ct}$ are the chosen variables of THz responses measured from the treated (T) area and control area (C) of skin at a specific time (t) point after removal of patches, and $X_{T0}$ and $X_{C0}$ are the variables measured from those two areas before patch application.

Trans-epidermal water loss is relatively constant in the longitudinal direction of the volar forearm [33]. Furthermore since we are calculating the NRC of variables, we are able to make meaningful comparisons between the patches in the different positions within the volar forearm.

## 3. Results and discussion

### 3.1 Variation on the original skin condition

**Fig. 4.**
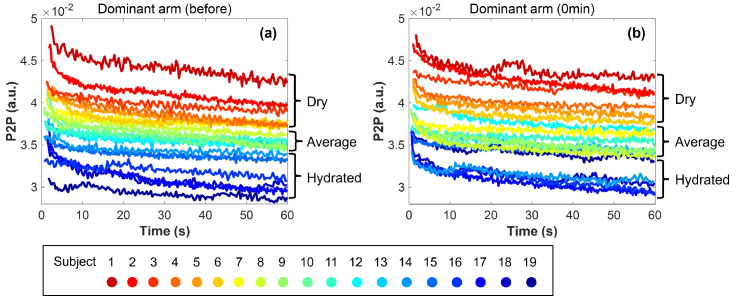
Occlusion curves of the control area on the dominant arm measured (a) before patch application and (b) 0 min after taking off the patches for all subjects.

Among the 19 subjects measured, variation can be seen in their original skin hydration profile without patch application. As illustrated in Fig. 4, the largest P2P variation between subjects is approximately 3-5 times the ΔP2P of one subject throughout the occlusion process for the control area on the dominant arm. As the P2P value is associated with the hydration level of skin in the way that lower P2P represents higher water content in skin, it is therefore important to categorize the subjects into different groups according to their original skin condition given by the control areas. Here we categorize them into three groups: subject 1-6 as the ‘Dry’ group; subject 7-13 as the ‘Average’ group; and subject 14-19 as the ‘Hydrated’ group. Figure 4(b) shows an example of the processed THz signals of all subjects measured at 2s into occlusion, providing more details on the P2P differences between each skin group. Figure 4 is a rough indication of the grouping, whereas the actual categorization is based on a comprehensive analysis of the hydration state of the control area of different subjects at several time points (before, 0 min, 30 min and 4 hours) and also their individual skin response to the patch treatments. The categorization is customized for the group of subjects in this study with a purpose of isolating the unusual results and studying the correlation between the unusual skin responses and the original skin condition. Further research needs to be conducted to give a robust method for the categorization of skin conditions in a general sense. In the following sections, we will be looking at the statistical trends for each group as well as for all subjects in general.

### 3.2 Effect of patch application on the skin hydration level

NRC of the sampled P2P is calculated for all subjects at 0 min, 30 min and 4 hours after the removal of the different patches as according to Eq. (2). A negative NRC value of P2P represents an increase in skin hydration level compared to the original state before patch application and a positive value implies a decrease; the hydration level is inversely proportional to the P2P NRC.

**Fig. 5.**
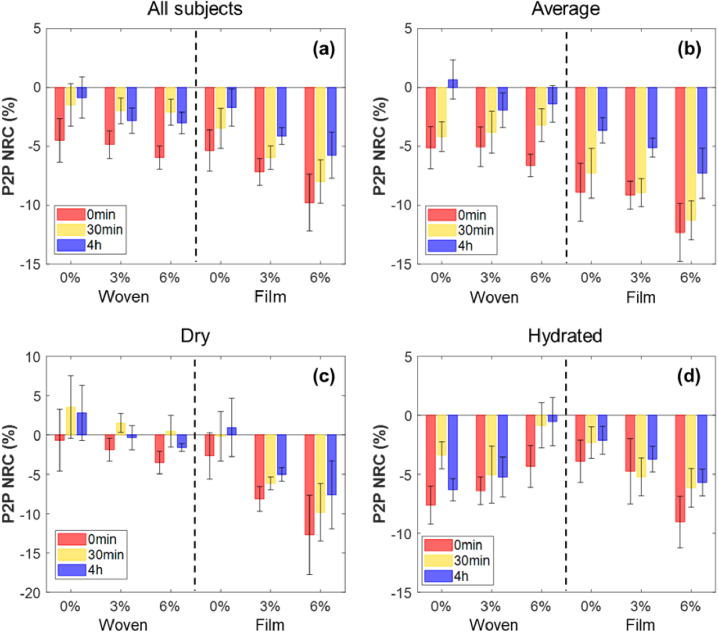
The average normalized relative change (NRC) of the sampled P2P in (a) all 19 subjects, (b) the ‘Average’ group, (c) the ‘Dry’ group and (d) the ‘Hydrated’ group measured at 0 min, 30 min and 4 hours after the removal of different patches (woven patches and film patches with 0%, 3% and 6% propylene glycol). Error bars are acquired by the standard error on the mean.

The average P2P NRC of all subjects is shown in Fig. 5(a) with error bars indicating the standard error in the mean. In general, an increase in skin hydration associated with the decrease in P2P is observed for all patch application areas at 0 min after the patches were removed after being applied for 24 hours. The changes show a declining trend with time indicating the recovery process of the skin, however the hydration effect persists even 4 hours after removal. Changes induced by the film patches are slightly larger than the woven patches and last longer in time. Different excipient compositions are observed to have some influences on both patches, and a higher PG concentration results in more hydrated skin.

Compared to the ‘All subjects’ group, results given by the ‘Average’ group in Fig. 5(b) show similar trend; more notable differences are observed for skin hydration changes induced by film and woven patches, with areas covered by the film patches being more hydrated than woven patches after 0 min, 30 min and 4 hours of removal respectively. For this skin group, we can still see a positive correlation between PG concentration and skin hydration level for both patches. For subjects with ‘Dry’ skin group, both film and woven patches have smaller impact on the THz response of skin compared to the ‘Average’ group; on the other hand, the skin recovery rate increases in general and some areas appear to be even dryer than the original state after 30 min following the removal of the patches, as shown in Fig. 5(c). When the original skin state of the subjects is more hydrated than average group, we can observe from Fig. 5(d) that film patches induce smaller changes on skin than the woven patches; and the recovery rate of the skin strongly decreases for all areas, leading to the skin staying at a highly hydrated state even 4 hours after removing the patches. A possible explanation is that when the original skin is already hydrated to a certain extent, occlusion effect caused by the fully occlusive film patches is no longer dominant in altering the skin hydration level, while the partially occlusive woven patches allow moisture exchange between skin and air and have more effect on further hydrating the skin. The impact induced by different PG concentrations for the ‘Dry’ group and ‘Hydrated’ group shows similar trend as observed in all subjects.

### 3.3 Effect of patch application on the skin occlusion process

The study shown in the last section provides an idea on how patch application interacts with the general hydration level in skin. Due to the increased hydration levels of the skin after the application of the patches, it is natural to ask if the skin’s response to occlusion has also been affected. As mentioned in Fig. 3, the occlusion process happens on untreated skin once it is in contact with the imaging window, blocking the exchange of water with the environment during the one-minute measurement. This process is also observed as a decrease in the P2P of the THz waveform which can be modelled by a biexponential curve. However, if the patch application disrupts the normal water distribution in skin, changes in the occlusion curve would be expected and the defined ΔP2P can be used as a variable for quantification.

**Fig. 6.**
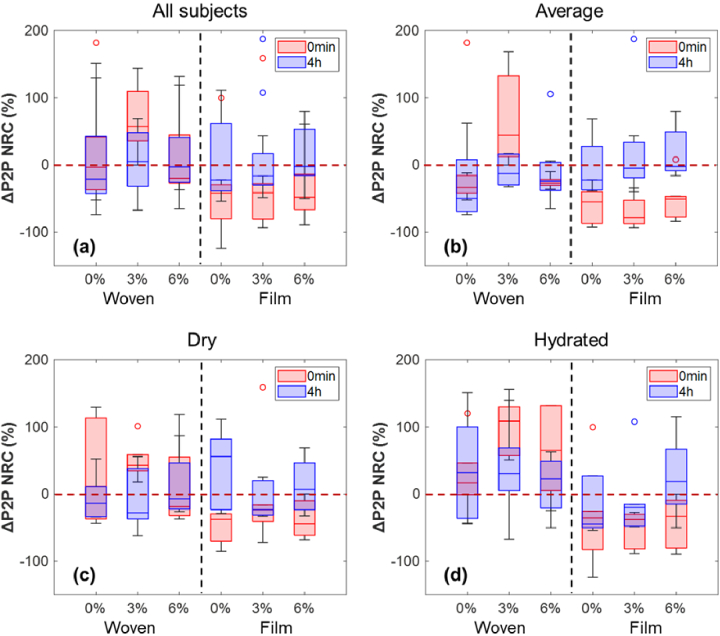
Box plots showing the NRC of ΔP2P in (a) all 19 subjects, (b) the ‘Average’ group, (c) the ‘Dry’ group and (d) the ‘Hydrated’ group measured at 0 min and 4 hours after the removal of different patches. The red/blue lines inside the boxes represent the median response; the upper and lower edges of the boxes are the upper and lower quartiles of the responses.

Figure 6 presents the NRC of ΔP2P in a box plot, where the red/blue lines inside the boxes indicate the median response and the upper and lower limits show the upper and lower quartiles. A negative NRC in ΔP2P suggests a decline in the variation of P2P during the occlusion process and that the occlusion curve flattens, which can imply that the skin is in a comparable condition of already being occluded and that further occlusion has less impact on it. While a positive NRC in ΔP2P is correlated with an increment in the change of P2P and the occlusion curve steepens.

As shown in Fig. 6(a-b), the ‘Average’ group presents similar results as taking all subjects into account, where looking at the film patches at 0 min after removal we can see ΔP2P NRC far below the zero-line indicating flattened occlusion curves. This observation further demonstrates that the impact on skin hydration by film patches is through the fully-occlusive feature. Woven patches, on the other hand, seem to have different effect on skin occlusion for different PG concentrations. After 4 hours of removing the patches, ΔP2P NRC for all excipient concentrations goes back to the state before treatment, revealing a recovery process for the water distribution in skin. For the ‘Dry’ group in Fig. 6(c), the impact of the film patches on skin occlusion decreases compared to the ‘Average’ group and barely any impact is seen for woven patches. In Fig. 6(d), we can still observe clear occlusive effect for all the film patches; this indicates that for skin that is already much hydrated, film patches still have an occlusive effect on it although they do not increase the skin hydration level as much (see Fig. 5(d)).

### 3.4 Statistical significance

**Fig. 7.**
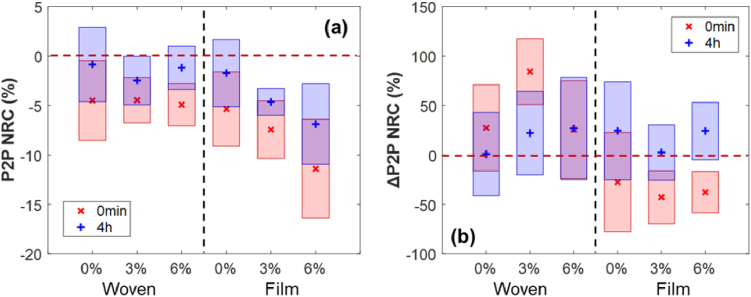
Results of performing the one-way analysis of variance test on (a) the NRC of sampled P2P and (b) the NRC of ΔP2P for all subjects measured at 0 min and 4 hours after the removal of patches. The cross/plus markers indicate the estimated mean of distribution; the shaded bars show the 95% confidence intervals calculated with the Dunnett’s test. The control group is represented by the red dashed line at the value of 0.

In addition to the data analysis in the previous sections, the statistical significance of the changes in skin hydration level and skin occlusion process induced by patch application is tested with the one-way analysis of variance (ANOVA) test and the post hoc Dunnett’s test among all the 19 subjects. The NRC of P2P measured at 0 min and 4 hours after removal of 6 types of patches is tested along with the control group which has an NRC value of 0 to check if there is significant difference after treatment. According to the one-way ANOVA test there is a statistically significant difference between groups for both measurement times, and Fig. 7(a) illustrates the results from the Dunnett’s test where the shaded bars represent the 95% confidence intervals. It is observed from Fig. 7(a) that all patches result in a significant change in the P2P of skin at 0 min, while the changes induced by 3% and 6% film patches persist significant after 4 hours. The NRC of ΔP2P is tested in a similar pattern and results are presented in Fig. 7(b). The one-way ANOVA test reveals statistically significant difference between groups for those measured at 0 min; from the Dunnett’s test, the 3% and 6% film patches and the 3% woven patch have significant impact on the ΔP2P of skin immediately after removing the patches, and all changes lose their significance after 4 hours.

### 3.5 Visualization of changes on skin hydration and surface profile

**Fig. 8.**
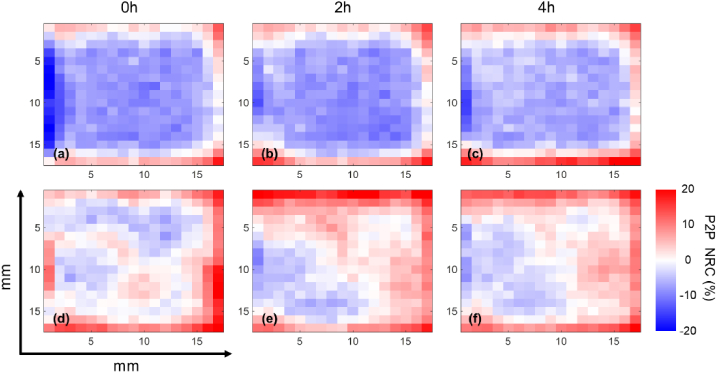
THz imaging results of the skin areas of one subject measured at 0 h, 2 h and 4 hours after the removal of (a-c) 6% film patch and (d-f) 6% woven patch.

THz imaging is performed for better visualization of the hydration change in the entire region of skin covered by patches. Figure 8 shows the imaging results of the skin areas of one subject from the ‘Average’ group at different times after removing the 6% film patch (Fig. 8(a-c)) and the 6% woven patch (Fig. 8(d-f)). In general, blue areas indicate negative NRC in P2P, associated with an increase in the skin hydration level. We can conclude from the figures that the film patches compared with the woven patches have greater impact on the skin with a larger hydrated area and a deeper hydration level. The skin area applied with film patches stay hydrated for at least 4 hours with the hydration level only decreasing slightly. This imaging result serves as a complement to the quantitative results in Fig. 5, showing the impact of patches on the entire treated area of skin with more spatial information.

**Fig. 9.**
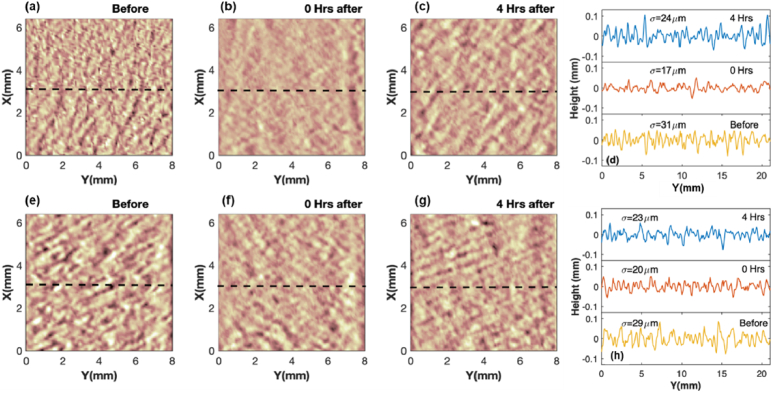
3D camera images of the skin area of one subject taken before patch application, 0 min and 4 hours after removing (a-c) the 6% film patch and (e-g) the 6% woven patch. Roughness of the skin surface at different times with the application of (d) the 6% film patch and (h) the 6% woven patch. Roughness is acquired along the black dashed lines on 3D images. Standard deviation σ is calculated.

The 3D camera is able to take images of an object obtaining the height information in the z-axis along an x-y surface, which in this study can be used to measure the roughness of the skin surface as shown in Fig. 9. Our previous discussions indicate that film patches hydrate the skin through occlusion effect, and according to Fig. 8 the hydration is more spatially uniform and persistent than that induced by woven patches. Therefore we used the 3D camera to measure the skin area of one subject from the ‘Average’ group before and after treatment with the 6% film patch and the 6% woven patch. In Fig. 9(a-c), we can directly see the change in skin roughness before and after the application of the film patch; the skin surface is clearly smoother immediately after the patch removal, and its roughness recovers partially after 4 hours yet remains less bumpy than before. To quantify the change in the surface roughness, height information along the white dashed line in each 3D image is acquired and the standard deviation is calculated, where smaller standard deviation is correlated with a smoother surface. From Fig. 9(d) we can see that before treatment σ=31μm , while 0 min after removing the film patch σ decreases to 17μm , and then after 4 hours it goes back to 24μm . In contrary, the skin treated with the woven patch does not have such a visible difference in the surface roughness before and after treatment, as shown in Fig. 9(e-g). The quantitative evaluation of the roughness in Fig. 9(h) indicates that the standard deviation σ changes from 29μm to 20μm immediately after removing the woven patch and then increases to 23μm after 4 hours.

3D images can display the roughness changes in skin surface before and after patch application with a straightforward visualization. Results show that occlusion induced by film patches changes the skin surface profile by smoothing the surface and reduces the depth of furrows, while accumulating water in the stratum corneum [34].

## 4. Summary

In this work, we have used *in vivo* THz spectroscopy to quantify the changes in human skin induced by transdermal drug delivery patches with different backing materials and propylene glycol concentrations. We found that the influence of patches on skin hydration and skin’s response to occlusion depends on the initial skin type of the subject as well as the patch backing and excipients used. Patches with a film backing increased the hydration in all but the most hydrated skin subjects, and this effect dominated over the concentrations of excipients used. Patches with a woven backing did not increase the hydration as much and the effect of the concentration changes in excipients was noticeable across most subjects. We have shown how THz sensing can be used to quantitively evaluate the direct impact on skin along with the resultant recovery process caused by different patches. THz imaging and 3D camera imaging complemented the results. Our work provides further insights into the impact of different types of TDD patches on skin and the mechanism behind it. The results demonstrate the potential application of *in vivo* THz sensing in the design and evaluation process of patches for transdermal drug delivery.

## Data Availability

Data underlying the results presented in this paper are available in Ref. [35,36].
